# The traces of imagination: early attention bias toward positively imagined stimuli

**DOI:** 10.1007/s00426-022-01737-0

**Published:** 2022-09-20

**Authors:** Hannah E. Bär, Jessica Werthmann, Andreas Paetsch, Fritz Renner

**Affiliations:** Department of Clinical Psychology and Psychotherapy, Institute of Psychology, University of Freiburg, Freiburg, Germany

## Abstract

Positively imagined activities may capture visual attention due to an increase in positive value. Increasing attention toward activities, in turn, may prove useful for clinical interventions aiming to motivate behavioral engagement. Employing a within-subject experimental design, we examined the effect of positive imagery on attention using a visual probe task with concurrent eye tracking. Adults from the general population (*N* = 54) imagined performing activities involving visually presented objects in a positive (focusing on the positive emotional impact) or neutral (focusing on a neutral circumstance) manner. They then completed a visual probe task using picture stimuli depicting one object per type of imagery. Positive compared to neutral imagery increased self-reported behavioral motivation and biased the direction, but not the duration, of gaze toward objects associated with the imagined activities. An exploratory analysis showed a positive association between the direction bias and depressive symptoms. Our findings build on existing literature on positive imagery as a *motivational amplifier* by highlighting early attention as an underlying cognitive mechanism.

**Supplementary Information:**

The online version contains supplementary material available at 10.1007/s00426-022-01737-0.

## Introduction

To imagine our future can be a source of motivation. Mental imagery, an internally generated perceptual experience, has long been thought to drive and guide behavior by representing a desired goal (McMahon, 1973). In the treatment of psychological disorders, these properties of mental imagery may prove useful to promote adaptive behaviors (Renner et al., 2021). By imagining the positive emotional impact of prospective activities, reward anticipation may be strengthened and, in turn, motivate behavioral engagement (Renner et al., 2021; Rösch et al., 2021).

Previous work has shown that mental imagery has the capacity to evoke emotions on a subjective, physiological and neural level (Holmes & Mathews, 2010; Ji et al., 2016). Mental imagery relies on similar neural processes as perception and, thus, is thought to function as a simulation of reality inducing similar emotional and behavioral responses (Dijkstra et al., 2019, 2021; Ji et al., 2016; Lang, 1978; Sambuco et al., 2020). Indeed, multiple experiments have demonstrated that imagining emotional (positive and negative) events increases emotional engagement more than imagining neutral events (Brandi et al., 2021; Henderson et al., 2018) or thinking about emotional events verbally (Holmes et al., 2006, 2008; Nelis et al., 2015). On a physiological level, mental imagery of emotional compared to neutral events further has been associated with enhanced pupil dilation, reflecting higher levels of emotional arousal (Henderson et al., 2018). This is mirrored by evidence suggesting that the inability to perform visual imagery while reading emotional imagery-provoking scenarios (i.e. verbal-focused processing) is related to a lack of emotional arousal, evidenced by an attenuated skin conductance response (Wicken et al., 2021). Likewise, neural activation in emotion processing brain regions, such as the ventromedial prefrontal cortex and striatum, has been shown to scale with the affective value of imagined experiences (Benoit et al., 2014; Lee et al., 2021). Together, these findings highlight a unique relation between mental imagery and emotion. Imagining the positive emotional impact of engaging in activities, thus, may be well suited to amplify behavioral motivation.

Recent studies have focused on the use of positive imagery to promote behavioral motivation. In these studies, individuals were asked to schedule and then imagine performing everyday activities, focusing on the most positive aspects of the activities (Heise et al., 2022; Ji et al., 2021; Renner et al., 2019). Compared to individuals who were asked to only schedule activities (without mental imagery) or to imagine the process of performing activities (without a focus on positive aspects), individuals who positively imagined performing the activities reported greater anticipatory pleasure (i.e. pleasure experienced when imagining the activity), anticipated pleasure (i.e. pleasure expected to be experienced when performing the activity) and behavioral motivation. However, in contrast to performing a verbal-reasoning task, mental imagery only increased anticipatory pleasure but not anticipated pleasure and behavioral motivation (Ji et al., 2021). Further support for the motivational effect of mental imagery is provided by Hallford et al. (2020), who showed that nominating and imagining upcoming positive events enhances ratings of pleasant anticipation and behavioral intention relative to baseline. A study by Linke and Wessa (2017) corroborates these findings on a more implicit level. Compared to a waitlist control condition, the authors found faster approach tendencies toward positive activities following a 2-week mental imagery training during which participants imagined activities they found personally enjoyable. This also fits well with the work by Oettingen (2014) showing that imagining personal goals and how to achieve them increases behavioral motivation. Thus, initial evidence indeed suggests that positive imagery of everyday activities promotes behavioral motivation.

To use the increase in behavioral motivation through positive imagery to promote adaptive behaviors effectively, a more in depth understanding of the involved cognitive mechanisms is important. For behavioral motivation to give rise to motivated behavior, activities first need to be attended to. The role of attention in motivated behavior, however, has not yet been thoroughly investigated in this context. A meta-analytic review by Pool et al. (2016) showed that positive stimuli attract attention especially during the early stages of attentional processing. Also referred to as motivated attention (Lang et al., 1997), this type of attention bias has been seen for positive stimuli as well as for neutral stimuli with an acquired positive value (Pool et al., 2016). The latter, for instance, has been shown to increase visual search efficiency when it serves as the target and to decrease it when it serves as the distractor (Anderson & Yantis, 2012; Kristjánsson et al., 2010). Interestingly, visual attention can be biased regardless of whether stimulus-reward pairings are previously trained or merely instructed without additional training (Tibboel & Liefooghe, 2020). Paulus et al. (2022) further demonstrated that vividly imagining an interaction with liked or disliked people at neutral places shapes individuals’ attitudes toward the places in the respective direction. This imagery-based evaluative conditioning (Hofmann et al., 2010) may also take place during positive imagery of prospective activities, during which activities are associated with positive emotions. Positively imagined activities could, thus, be expected to gain attentional priority due to their increase in positive value.

### The present study

In the present study, the influence of positive versus neutral imagery of everyday activities on motivated attention was tested using a within-subject experimental design. Participants viewed pictures of objects while listening to brief audio recordings guiding the mental imagery of performing an activity involving the objects in either a positive or neutral manner. Attention bias for objects associated with positive imagery was then measured as a proxy of motivated attention using a visual probe task with concurrent eye tracking. Additionally, subjective ratings of the vividness and motivational impact (reward anticipation and motivation) of the mental imagery were recorded. Our preregistered hypotheses (https://osf.io/x4wvk) were: (1) Self-reported motivational impact (anticipatory reward, anticipated reward and motivation) will be greater for positive compared to neutral imagery, (2) attentional bias (direction and duration bias) will be greater for picture stimuli previously associated with positive compared to neutral imagery and (3) greater self-reported motivational impact of positive compared to neutral imagery will be positively associated with the attentional bias (direction and duration bias) toward picture stimuli from positive imagery trials.

## Method

### Participants

A total of 54 participants (42 female) between the age of 18 and 38 years (*M* = 23.00, *SD* = 4.15) were recruited from the German general public via online advertising and a community volunteer web portal. Educational achievement levels included high school (74.1%), university or higher (20.4%) and apprenticeship (5.6%). Eligible participants fulfilled the following criteria: (1) age between 18 and 65 years, (2) German language proficiency (native speaker or C1 CEFR level), (3) no diagnosis or treatment of a mental disorder within the last 6 months and (4) normal or corrected-to-normal vision and hearing. Participation was compensated with 10€ or course credit. The study was approved by the ethics committee of the German Psychological Society (2020-02-13VA).

### Procedure

Eligible participants entered the study after they received study information and provided (written) informed consent. Prior to the experimental session, they completed a short battery of online self-report questionnaires. Upon arrival at the eye tracking laboratory, participants first indicated their current affective state on a self-report questionnaire. They then received a brief introduction to mental imagery and subsequently completed an experimental paradigm, the Positive Imagery Paradigm (PIP), in which the valence of mental imagery was manipulated (positive versus neutral). This was followed immediately by a visual probe task to assess attention bias for stimuli associated with positive imagery during the PIP. The study was conducted in line with the COVID-19 guidelines of the Institute of Psychology of the University of Freiburg. Participants and experimenters wore FFP-2 facemasks throughout the entire experiment.

### Positive imagery paradigm

The PIP was developed based on a similar paradigm by Holmes et al. (2008) to experimentally manipulate motivation to perform an activity through mental imagery. Participants were shown picture stimuli of objects (e.g. a bathtub) on a computer screen while simultaneously listening to an imagery script guiding the mental simulation of performing an activity involving the depicted object in a positive (positive imagery [PI] condition) or neutral (neutral imagery [NI] condition) manner.

The PIP comprised 30 trials equally divided across conditions. All trials began with a fixation cross displayed for 1000 ms, followed by the (visual and auditory) presentation of the individual objects and corresponding imagery scripts all varying between 10 and 13 s. Subsequently, self-reported vividness and motivational impact of the mental imagery were measured (see “Questionnaires and rating scales”). The pairing of an object with PI or NI was counterbalanced so that one half of the participants imagined an activity in a positive manner, while the other half of participants imagined the same activity in a neutral manner. The order of trials was randomized across participants.

#### Imagery scripts and picture stimuli

Imagery scripts guiding the mental simulation of performing an activity were developed for the purpose of the present study. All scripts were three sentences long and followed the same basic structure: (1) place and activity (e.g. *You are at home and draw yourself a bath.*), (2) action involving an activity-related object (e.g. *You get into the bathtub and lean back.*) and (3) positive emotional reaction to the activity (e.g. *The warm water lets you relax completely.*; PI condition) or description of a neutral circumstance of the activity (e.g. *A foam layer covers the water surface*; NI condition). Activities were chosen based on the assumption that they are familiar to a large number of people (e.g. taking a bath or going for a run) to ensure the accessibility of activity-related detail to form a vivid mental image. Half of the audio-recorded imagery scripts were spoken by a male speaker and half by a female speaker. Picture stimuli of objects mentioned in the imagery scripts were used under license from Shutterstock.com. The imagery script and picture pairs were finalized after initial pilot testing.

### Visual probe task

**Fig. 1:**
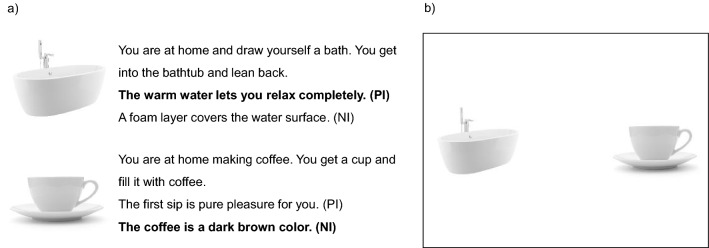
Illustration of the experimental paradigm depicting **a** the Positive Imagery Paradigm (example of counterbalanced conditions with conditions in bold presented to participant A and conditions in normal font presented to participant B and **b** the subsequent visual probe task (pictures used under license from Shutterstock.com)

A visual probe task (MacLeod et al., 1986) with concurrent eye tracking was used to measure attention bias for objects associated with positive imagery. Image pairs depicting two objects from the PIP were presented next to each other on a computer screen (see Fig. 1). Each image pair consisted of one object previously imagined in a positive manner (e.g. the bathtub in Fig. 1) and one object previously imagined in a neutral manner (e.g. the coffee cup in Fig. 1) during the PIP. This was followed by the presentation of a probe that replaced one of the objects. Participants were instructed to indicate the probe’s location as fast as possible with a corresponding key press.

The task comprised 60 trials randomized across participants. All trials started with a fixation cross presented until participants fixated their gaze on it for 100 ms. This was followed by the presentation of the image pairs for 3000 ms and the subsequent probe until participants responded (self-paced). The position of the objects and probe was counterbalanced so that each image pair was presented four times. All image pairs were matched on visual aspects, such as color and complexity.

The present study primarily focused on eye movements recorded during the object pair presentations as a direct measure of attention rather than reaction times as an indirect measure of attention (presuming faster responses to probes replacing stimuli of attention). The task recently demonstrated good internal consistency and test–retest reliability of eye tracking-based attention bias for rewarding stimuli (i.e. food; van Ens et al., 2019).

### Eye tracking

To assess attention allocation during the visual probe task, eye movements (i.e. gaze fixations) were recorded with a desktop mounted EyeLink 1000 Plus system (SR Research Ltd., Mississauga, Ontario, Canada) following a 9-point calibration and subsequent validation procedure. Gaze fixations were defined as any period of at least 100 ms that is not a blink or saccade. Fixation data for periods of object pair presentations were divided into three interest areas, including the midsection (i.e. prior fixation cross location) and the left and right sections (i.e. object locations) of the presentation screen. Following standard procedures, fixations located on the midsection of the presentation screen and anticipatory fixations were discarded. Participants identified as “starer” (i.e. below average [minus two standard deviations] gaze dwell time on PI or NI objects and/or no fixation on objects in more than 50% of trials) were excluded from analyses (Werthmann et al., 2016). Based on this, eye tracking data of four participants were removed from analyses. The adjusted sample size of 50 participants for eye tracking data analyses yielded a power of 93.4% for an effect size of *d* = 0.5 and *α* = 0.05.

### Attention bias

Early (direction bias) and sustained (duration bias) attention allocation (Werthmann et al., 2016) toward PI objects was assessed per participant. A direction bias is indicated by a greater proportion of trials in which the first fixation is located on PI objects relative to NI objects (Werthmann et al., 2016). To calculate a direction bias score, the number of trials with the first fixation on PI objects is divided by the sum of all trials with an initial fixation on one of the objects. A score above 50% indicates a direction bias toward PI objects. A duration bias is suggested by a longer average gaze dwell time (in ms) on PI objects compared to NI objects (Werthmann et al., 2016). To compute a duration bias score, the mean gaze dwell duration on PI objects is subtracted from the mean gaze dwell duration on NI objects. A positive score suggests a duration bias for PI objects.

### Questionnaires and rating scales

#### Self-reported motivational impact and vividness

Self-reported motivational impact (*It was nice to imagine the activity* [anticipatory reward]; *I would enjoy the activity* [anticipated reward]; *I am motivated to perform the activity* [motivation]) and vividness (*I imagined the activity vividly*) of the mental imagery performed during the PIP were measured on continuous rating scales ranging from 0 (“not at all”) to 100 (“completely true”) following each trial. The self-report questions on anticipatory reward, anticipated reward and motivation were based on prior research (e.g. Heise et al., 2022; Ji et al., 2021; Renner et al., 2019).

#### Depression Anxiety and Stress Scale (DASS-21)

The DASS-21 (Henry & Crawford, 2005; Nilges & Essau, 2015) comprises 21 statements on depression, anxiety and stress over the past week. It is rated on a four-point scale from 0 (“did not apply to me at all”) to 3 (“applied to me very much or most of the time”). In the present study, the depression subscale had good internal consistency (*α* = 0.82; *ω*_T_ = 0.83).

#### Positive and Negative Affect Schedule State Version (PANAS)

The PANAS (Krohne et al., 1996; Watson et al., 1988) assesses current positive and negative affect with 10 adjectives rated on a five-point scale from 1 (“not at all”) to 5 (“extremely”). In the present study, the internal consistency for the PA subscale was *α* = 0.84 and *ω*_T_ = 0.85 and for the NA subscale *α* = 0.62 and *ω*_T_ = 0.66.

#### Spontaneous Use of Imagery Scale (SUIS)

The SUIS (Görgen et al., 2016; Nelis et al., 2014) is a 12-item measure of everyday imagery use rated on a five-point scale ranging from 1 (“never”) to 5 (“always”). In the present study, the scale had good internal consistency (*α* = 0.88; *ω*_T_ = 0.89).

#### Plymouth Sensory Imagery Questionnaire (PSI-Q)

The PSI-Q (Andrade et al., 2014) consists of 35 items rated on a 11-point scale from 0 (“no image at all”) to 10 (“image as clear and vivid as real life”) assessing the vividness of visual, sensory and emotional mental imagery. In the present study, the scale had good internal consistency (*α* = 0.91; *ω*_T_ = 0.92).

### Data analysis

Paired sample t-tests were conducted to test differences in self-reported motivational impact (anticipatory reward, anticipated reward and motivation) between PI and NI (hypothesis 1) and differences in attention bias indices (direction and duration bias) between PI objects and NI objects (hypothesis 2). Pearson correlations were applied to explore the relationship between attention bias indices and self-reported motivational impact (hypothesis 3). A post-hoc exploratory correlation analysis was conducted between attention biases (direction and duration bias) and depressive symptoms as assessed with the DASS-21 depression subscale. Data from other questionnaires were not included in statistical analyses.

## Results

### Self-reported motivational impact and vividness

**Fig. 2:**
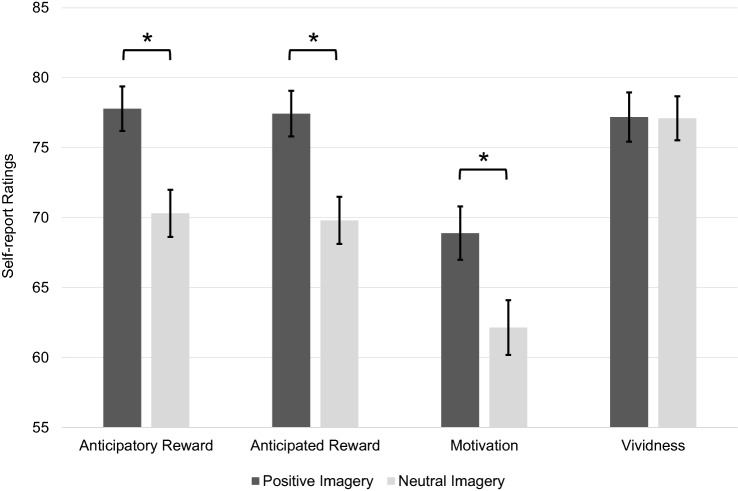
Self-reported motivational impact (anticipatory reward, anticipated reward and motivation) and vividness of positive versus neutral imagery of activities (original scale from 0 to 100) with error bars representing standard errors and asterisks indicating significant results (all *p* < 0.001 inspected at Bonferroni-corrected *α* of 0.017)

Participants reported significantly greater anticipatory reward (*t*(53) = 7.34, *p* < 0.001, *d* = 1.00), anticipated reward (*t*(53) = 6.46, *p* < 0.001, *d* = 0.88) and motivation (*t*(53) = 4.96, *p* < 0.001, *d* = 0.68) in response to PI (*M* = 77.79, *SD* = 11.70, *M* = 77.44, *SD* = 11.97 and *M* = 68.90, *SD* = 14.03, respectively) compared to NI (*M* = 70.31, *SD* = 12.40, *M* = 69.81, *SD* = 12.38 and *M* = 62.14, *SD* = 14.40, respectively).^1^ Results were inspected at a Bonferroni-corrected *α* = 0.017. PI (*M* = 77.19, *SD* = 12.92) and NI (*M* = 77.10, *SD* = 11.55) did not significantly differ in vividness (*t*(53) = 0.09, *p* = 0.928, *d* = 0.01) (Fig. 2).

### Attention bias for objects associated with positive imagery

**Fig. 3:**
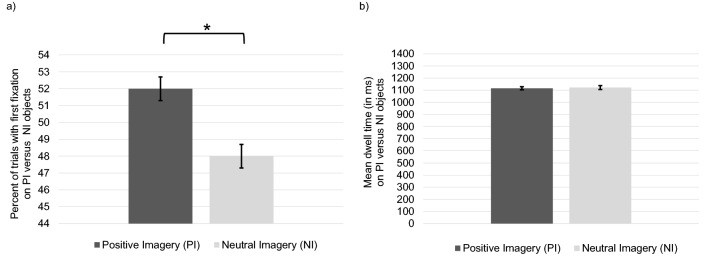
**a** Direction and **b** duration bias toward objects associated with positive versus neutral imagery with error bars representing standard errors and asterisks indicating significant results (*p* < 0.05)

Results showed a significant direction bias toward PI objects (*t*(49) = 2.18, *p* = 0.034, *d* = 0.31).^2^ Participants directed their initial gaze significantly more often on objects associated with PI (*M* = 0.52, *SD* = 0.05) compared to objects associated with NI (*M* = 0.48, *SD* = 0.05). Results did not show a significant duration bias for PI objects (*t*(49) = − 0.36, *p* = 0.720, *d* = − 0.05). Participants did not significantly differ in their gaze duration on objects associated with PI (*M* = 1116.10, *SD* = 91.41) compared to objects associated with NI (*M* = 1122.22, *SD* = 113.67) (Fig. 3).

### Relationship between attention biases and self-reported motivational impact

Pearson correlations were conducted between attention bias indices (direction and duration bias scores) and self-reported motivational impact (i.e. differences between anticipatory reward [*r* = − 0.14 and *r* = − 0.10, respectively], anticipated reward [*r* = − 0.16 and *r* = − 0.06, respectively] and motivation [*r* = − 0.19 and *r* = − 0.06, respectively] for PI versus NI). None of the inspected correlations were statistically significant (all *p* ≥ 0.20).

### Post-hoc exploratory correlation analyses

Post-hoc exploratory correlation analyses revealed that depressive symptom severity (DASS-21 depression sum-score) correlated moderately positively with the direction bias (*r* = 0.40, *p* = 0.004),^3^ but not with the duration bias (*r* = 0.02, *p* = 0.903).

## Discussion

This study examined the influence of positive imagery of everyday activities on attention using a visual probe task with concurrent eye tracking. Results showed that positive compared to neutral imagery increases self-reported behavioral motivation and biases the direction, but not the duration, of gaze on objects associated with the imagined activities. A post-hoc analysis revealed that the observed direction bias was more pronounced in individuals with greater depressive symptom severity. However, no evidence was found to suggest that the direction bias was related to subjective reports of behavioral motivation.

Imagining the positive emotional impact of performing an activity relative to a neutral circumstance of the activity shifted early attention, but not sustained attention, toward objects involved in the imagined activity. This is in line with Pool et al. (2016), who showed that positive stimuli attract attention especially during the early stages of attentional processing. It has been suggested that this is because desired stimuli attract attention automatically without conscious awareness (Frankland et al., 2016; Theeuwes, 2010; Theeuwes & Belopolsky, 2012). The lack of evidence for sustained attention on positively imagined objects, thus, could indicate that the positive imagery manipulation was not strong enough to influence a conscious evaluation of the stimuli at a later point in time. As predicted, however, positively imagined objects appear to have captured early attention due to an increase in positive value. This is in line with recent work suggesting that mental imagery can affect the evaluation of real-life environments (Benoit et al., 2019; Paulus et al., 2022). Paulus et al. (2022), as mentioned previously, demonstrated that imagining an interaction with liked or disliked individuals at an initially neutral place changes a person’s attitude toward that place in the respective direction. This imagery-based evaluative conditioning (Hofmann et al., 2010) could, thus, explain the present findings. The positive valence of emotions experienced during positive imagery (unconditioned stimulus) might have transferred to the simultaneously depicted objects (conditioned stimulus), thereby increasing their saliency during the subsequent visual probe task. Activities imagined in a positive compared to a neutral manner, indeed, were evaluated more positively by participants. They gave higher ratings of reward experienced during the positive imagery as well as higher ratings of anticipated reward and motivation to perform the activities. However, no evidence was found to suggest that these subjective reports were associated with the observed bias in initial orientation. It has been previously argued that early attention as opposed to sustained attention occurs independent of conscious awareness (Frankland et al., 2016; Theeuwes, 2010; Theeuwes & Belopolsky, 2012). In keeping with this, self-report measures assessing the reflection of performance (explicit processing) and behavioral measures assessing performance itself (implicit processing) do not always correspond (Dang et al., 2020). This could explain why behavioral motivation and early attention were not related in the present study. However, temporary goals have been argued to influence automatic attention processes (Vogt et al., 2020). A recent meta-analytic review by Hardman et al. (2021) further suggested that state motivation is a key determinant of attention bias for appetitive (food) stimuli. Contrary to this, in the present study, motivation was assessed specifically for each stimulus rather than as a general motivational state of the individual. Moreover, it is possible that the difference in inherent (food) versus acquired (objects associated with positive imagery) motivational value of stimuli might play an important role. It may also be that participants memorized objects imagined in a positive manner better than objects imagined in a neutral manner. Previous work, indeed, has shown a memory benefit for emotional compared to neutral material (Kensinger & Corkin, 2003). As reward associations can magnify the effect of memory on attention already in the early stages of perceptual processing (Doallo et al., 2013), the observed shift in early attention could also be explained by a better memory of positively imagined objects. Although more research is needed to fully understand the lack of relation between behavioral motivation and early attention in this context, this study highlights the potential of positive imagery to increase behavioral motivation and shift early attention toward activities through an increase in positive value.

Directing attention using positive imagery may prove useful to motivate the engagement in adaptive behaviors. Indeed, enhancing the saliency of (monetary) rewards has been recently found to increase motivated behavior in an effort-based decision-making task (Renz et al., 2021). Similarly, in real-life behavioral contexts, shifting early attention toward healthy food and lifestyle choices has been found to positively influence motivational approach (Suri & Gross, 2015). This is particularly interesting in light of clinical interventions aiming to promote behavioral activation. Here, positive imagery has been proposed as a *motivational amplifier* addressing reward anticipation deficits in depression (Renner et al., 2019, 2021). Previous research has suggested that these deficits in reward anticipation are the primary factor for reduced reward-motivated behavior (Bakker et al., 2017; Gorka et al., 2014; Sherdell et al., 2012). Moreover, depression has been associated with difficulties in the prioritization of environmental stimuli (Whitton et al., 2015). By shifting early attention toward environmental stimuli predictive of reward, positive imagery, thus, may be well suited to target these deficits in depression. Indeed, in the present study, individuals with greater depressive symptom severity were more susceptible to the positive imagery manipulation and showed a more pronounced shift in early attention. This further signifies the applicability of positive imagery in the context of depression.

### Strengths, limitations and future directions

A major strength of the present study is its within-subject experimental design with randomized and counterbalanced conditions. Moreover, imagery scripts were designed in such a way that only their valence changed according to the experimental condition. This way, differences on self-report and eye movement measures can be attributed to changes in valence rather than general content of the imagined activities. Future studies, however, are needed to examine whether the observed effects are amplified by the modality of thought (mental imagery) or merely represent the difference in valence (positive versus neutral). It would also be important to further explore the mechanism driving the effect of positive imagery on motivated attention and whether the effect is also present for mental representations of activities. Additionally, findings need replication in clinically depressed individuals to solidify the exploratory finding of a greater susceptibility to positive imagery in this patient group.

## Conclusion

The present study elucidates early attention as a cognitive mechanism affected by positive imagery. As early attention plays an important role in motivated behavior, applying positive imagery to increase the saliency of adaptive behaviors in the environment may be a valuable instrument to promote behavioral activation. One potential target group for this treatment are individuals with depressive symptoms, as they appear to be especially susceptible to positive imagery.

## Supplementary Information

Below is the link to the electronic supplementary material.

Supplementary file1 (DOCX 312 KB)

## Data Availability

The datasets generated during and/or analyzed during the current study are available from the corresponding author on reasonable request.

## Footnotes

1 If anticipatory reward, anticipated reward and motivation are treated as one concept of ‘motivational impact’ (internal consistency of α = .88 and *ω*_T_ = 0.88) *t*(53) = 6.56, *p* < 0.001, *d* = 0.89.

2 If no participant was excluded from the analysis *t*(53) = 2.12, *p* = .038, *d* = 0.29.

3 If controlled for anticipatory reward, anticipated reward and motivation *r* = 0.39, *p* = 0.007.
