# Supplementary material for: The traces of imagination: early attention bias toward positively imagined stimuli
Source: Psychol Res. 2022 Sep 20;87(5):1475–83. doi: 10.1007/s00426-022-01737-0 (PMC9485787; doi:10.1007/s00426-022-01737-0)
Supplement: Supplementary file 1 — Supplementary file1 (DOCX 312 KB) [file 426_2022_1737_MOESM1_ESM.docx]

**Supplementary Material**

**A: Methods**

**Table A1.** Study material used in the positive imagery paradigm and the visual probe task

| **Image Pair 1** | |
| --- | --- |
| 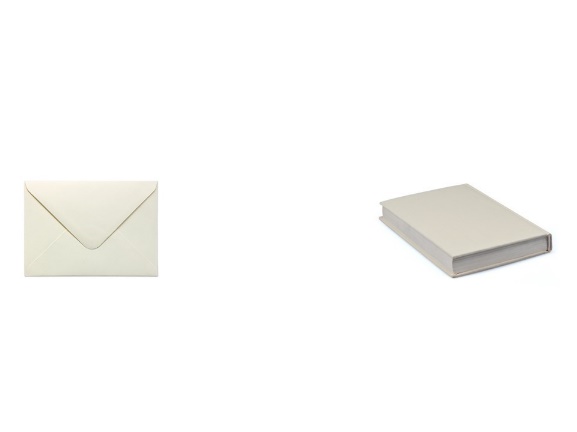 | |
| *English translation*  You are on your way sending a letter. You open the mailbox flap and post the letter.   - The joy that you will bring with it fills you with feelings of happiness. (PI) - According to the sign, the mailbox will be emptied this afternoon. (NI)   *German original*  Du bist unterwegs und verschickst einen Brief. Du öffnest die Briefkastenklappe und wirfst den Brief ein.   - Die Freude, die du damit bereiten wirst, erfüllt dich mit Glücksgefühlen. (PI) - Laut dem Schild wird der Briefkasten noch heute Nachmittag geleert. (NI) | *English translation*  You are sitting in the living room reading. You turn a page of your book and read the first few lines.   - The exciting scene awakens in you a great desire to read more. (PI) - The lines describe a brief dialogue between two characters. (NI)   *German original*  Du sitzt im Wohnzimmer und bist am Lesen. Du blätterst eine Seite deines Buches um und liest die ersten Zeilen.   - Die spannende Szene weckt in dir ein großes Verlangen weiterzulesen. (PI) - Die Zeilen beschreiben einen kurzen Dialog zwischen zwei Charakteren. (NI) |
| **Image Pair 2** | |
| 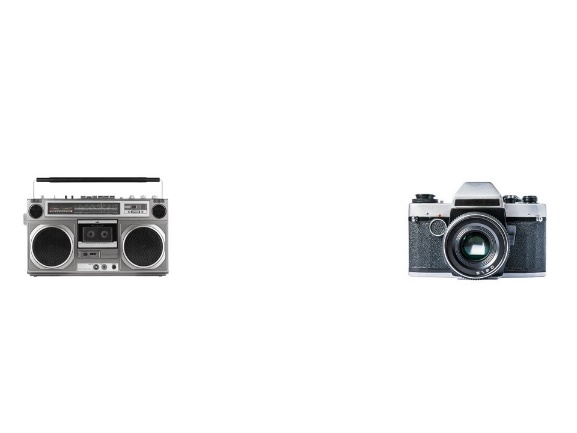 | |
| *English translation*  You are at home listening to music. You switch the radio station and adjust the volume.   - With great enthusiasm, you hear the first notes of your favorite song. (PI) - The traffic news are currently running on the newly tuned-in station. (NI)   *German original*  Du bist zuhause und hörst Musik. Du wechselst den Radiosender und stellst die Lautstärke ein.   - Mit großer Begeisterung hörst du die ersten Töne deines Lieblingsliedes. (PI) - Auf dem neu eingestellten Sender laufen gerade die Verkehrsnachrichten. (NI) | *English translation*  You are out to take photos. You line up the camera and press the shutter button.   - The successful snapshot fills you with great pride. (PI) - The image shows a small group of trees. (NI)   *German original*  Du bist unterwegs, um zu fotografieren. Du richtest die Kamera aus und drückst auf den Auslöser.   - Der gelungene Schnappschuss erfüllt dich mit viel Stolz. (PI) - Die Bildaufnahme zeigt eine kleine Gruppe von Bäumen. (NI) |
| **Image Pair 3** | |
| 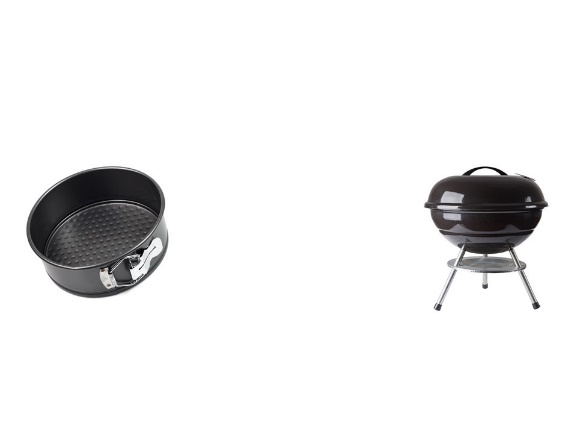 | |
| *English translation*  You are at home baking a cake. You put the cake pan in the oven and close the oven door.   - You cannot wait to finally enjoy the cake. (PI) - The oven is pre-heated to 160 degrees Celsius. (NI)   *German original*  Du bist zuhause und backst einen Kuchen. Du schiebst die Kuchenform in den Backofen und schließt die Ofentür.   - Du kannst es kaum abwarten, den Kuchen endlich zu genießen. (PI) - Der Backofen ist bei Umluft auf 160 Grad Celsius vorgeheizt. (NI) | *English translation*  You are in the garden having a barbecue with your family. You fill the barbecue with charcoal and light it.   - You are overwhelmed by a thrill of anticipation of the shared meal. (PI) - The charcoal takes on a whitish color due to the heat. (NI)   *German original*  Du bist im Garten und grillst mit deiner Familie. Du füllst den Grill mit Grillkohle und zündest sie an.   - Dich überkommt pure Vorfreude auf die gemeinsame Mahlzeit. (PI) - Die Grillkohle nimmt durch die Hitze eine weißliche Farbe an. (NI) |
| **Image Pair 4** | |
| 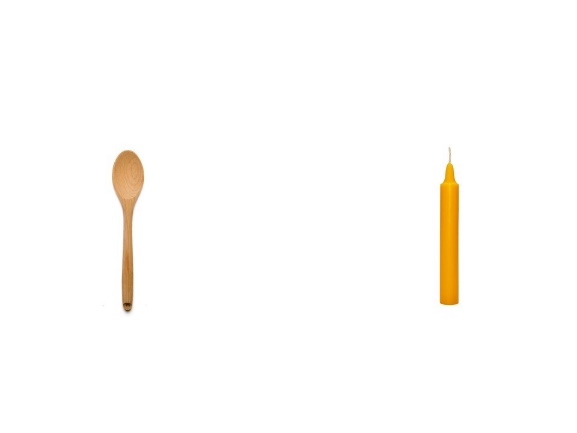 | |
| *English translation*  You are at home cooking dinner. You take a cooking spoon and stir the saucepan with it.   - You enjoy the growing anticipation of the meal. (PI) - A little steam rises from the saucepan. (NI)   *German original*  Du bist zuhause und kochst Abendessen. Du nimmst dir einen Kochlöffel und rührst damit den Kochtopf um.   - Du genießt die steigende Vorfreude auf das Essen. (PI) - Aus dem Kochtopf steigt etwas Wasserdampf auf. (NI) | *English translation*  You are at home having a candle light dinner. You light a candle and sit down at the table.   - The closeness to each other evokes a deep affection in you. (PI) - The table is set with plates, cutlery and glasses. (NI)   *German original*  Du bist zuhause auf einem Candle-Light-Dinner. Du zündest eine Kerze an und setzt dich an den Tisch.   - Die Nähe zueinander rührt eine tiefe Zuneigung in dir. (PI) - Der Tisch ist mit Tellern, Besteck und Gläsern gedeckt. (NI) |
| **Image Pair 5** | |
| 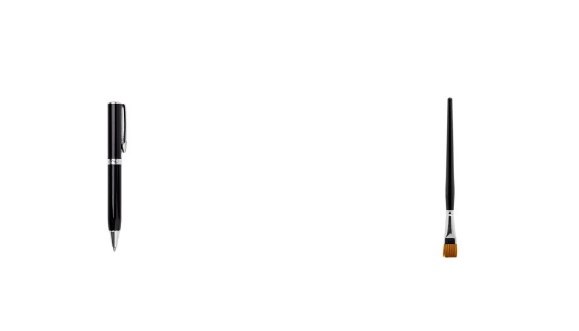 | |
| *English translation*  You are at home writing in your diary. You pick up a pen and start writing.   - You look back on the day with great satisfaction. (PI) - Every row is defined by a thin line. (NI)   *German original*  Du bist zuhause und schreibst in dein Tagebuch. Du nimmst einen Stift in die Hand und fängst an zu schreiben.   - Du blickst voller Zufriedenheit auf den Tag zurück. (PI) - Jede Zeile ist durch eine dünne Linie vorgegeben. (NI) | *English translation*  You are at home painting. You dip the brush into the paint and place it on the canvas.   - With every stroke of the brush, you are fully immersed in your creativity. (PI) - The motif is pre-sketched in pencil on canvas. (NI)   *German original*  Du bist zuhause am Malen. Du tauchst den Pinsel in die Farbe und setzt ihn auf der Leinwand auf.   - Mit jedem Pinselstrich gehst du ganz in deiner Kreativität auf. (PI) - Das Motiv ist auf der Leinwand mit Bleistift vorskizziert. (NI) |
| **Image Pair 6** | |
| 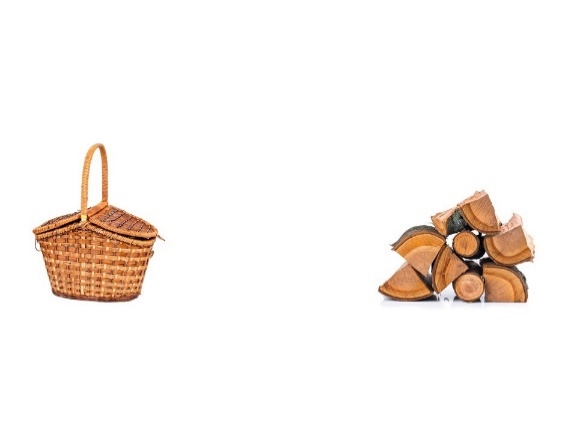 | |
| *English translation*  You are having a picnic with friends in the park. You put down the picnic basket and sit down on a blanket.   - The surrounding nature evokes spring fever in you. (PI) - The blanket has a typical red tartan pattern. (NI)   *German original*  Du bist mit Freunden im Park picknicken. Du stellst den Picknickkorb ab und setzt dich auf eine Decke.   - Die umliegende Natur weckt Frühlingsgefühle in dir. (PI) - Die Decke hat ein typisch rotes Schottenmuster. (NI) | *English translation*  You make a campfire with friends. You take some logs and put them on the fire.   - The cozy get-together gives you a feeling of security. (PI) - The fire is surrounded by a circle of stones. (NI)   *German original*  Du machst mit Freunden ein Lagerfeuer. Du nimmst ein paar Holzscheite und legst sie in das Feuer.   - Das gemütliche Beisammensein gibt dir Geborgenheit. (PI) - Das Feuer ist umgeben von einem Kreis aus Steinen. (NI) |
| **Image Pair 7** | |
| 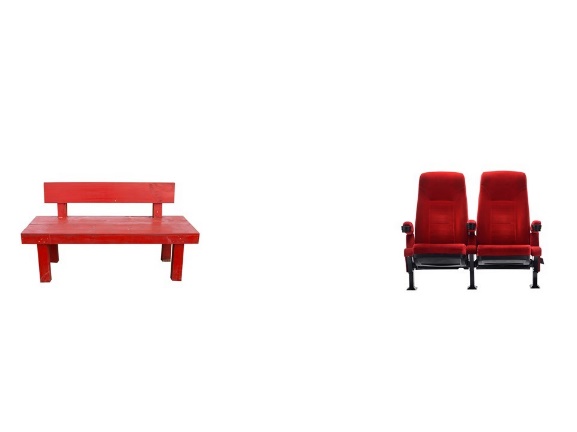 | |
| *English translation*  You are walking in the woods. You walk along a bend in the road and sit down on a bench at the edge of the forest.   - You are overwhelmed by the fantastic view. (PI) - The view is directed toward a spacious field. (NI)   *German original*  Du bist im Wald spazieren. Du gehst eine Wegbiegung entlang und setzt dich auf eine Bank am Waldrand.   - Du wirst überwältigt vom dem traumhaften Ausblick. (PI) - Der Ausblick ist auf ein weitläufiges Ackerfeld gerichtet. (NI) | *English translation*  You are at the movies with friends. You sit down in the chair and put down your belongings.   - You are looking forward to the movie with great anticipation. (PI) - There are already many other moviegoers in the big hall. (NI)   *German original*  Du bist mit Freunden im Kino. Du setzt dich in den Kinosessel und legst deine Sachen ab.   - Voller Vorfreude fieberst du der Filmvorführung entgegen. (PI) - In dem großen Saal sind bereits viele andere Kinobesucher. (NI) |
| **Image Pair 8** | |
| 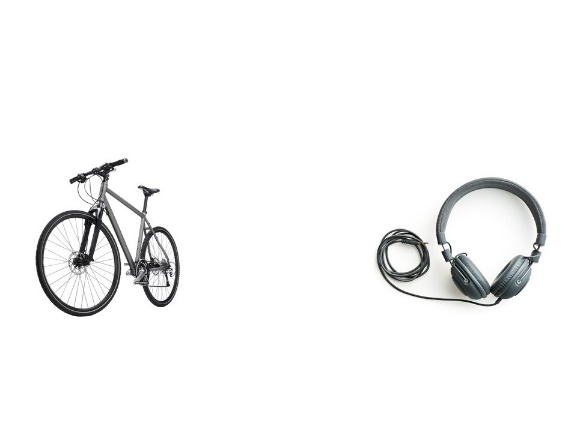 | |
| *English translation*  You are on a bike ride with friends. You ride down a straight road and pedal your bikes.   - The feeling of freedom awakens a pure joy of living in you. (PI) - The speed leads to a little more headwind. (NI)   *German original*  Du bist mit Freunden auf einer Fahrradtour. Ihr fahrt eine gerade Straße entlang und tretet in die Pedale eurer Räder.   - Das Freiheitsgefühl weckt eine pure Lebensfreude in dir. (PI) - Die Schnelligkeit führt dabei zu etwas mehr Gegenwind. (NI) | *English translation*  You are at home listening to a podcast. You grab headphones and listen to the conversation.   - You are totally carried away by the exciting topic. (PI) - The conversation is led by two scientists. (NI)   *German original*  Du bist zuhause und hörst einen Podcast. Du nimmst dir Kopfhörer und hörst der Unterhaltung zu.   - Du wirst total mitgerissen von dem spannenden Thema. (PI) - Die Unterhaltung wird von zwei Wissenschaftlern geführt. (NI) |
| **Image Pair 9** | |
| 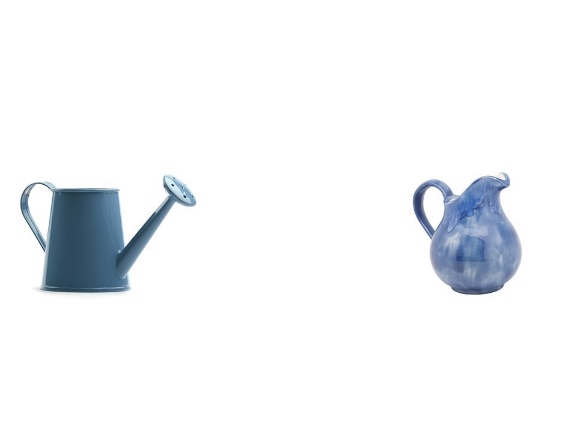 | |
| *English translation*  You are outside working in the garden. You reach for the watering can and water the herb garden.   - The magnificent sight fills you with great pride. (PI) - The water turns the earth a dark brown color. (NI)   *German original*  Du bist draußen und arbeitest im Garten. Du greifst nach der Gießkanne und bewässerst den Kräutergarten.   - Der prächtige Anblick erfüllt dich mit großem Stolz. (PI) - Das Wasser verfärbt dabei die Erde dunkel braun. (NI) | *English translation*  You are in a studio making pottery. You take a piece of clay and form a pitcher out of it.   - You are incredibly proud of your creative output. (PI) - The remains of clay are scattered all over the floor. (NI)   *German original*  Du bist in einem Atelier und töpferst. Du nimmst ein Stück Ton und formst daraus einen Krug.   - Du bist unheimlich stolz auf deine kreative Leistung. (PI) - Die Tonreste sind überall auf dem Boden verteilt. (NI) |
| **Image Pair 10** | |
| 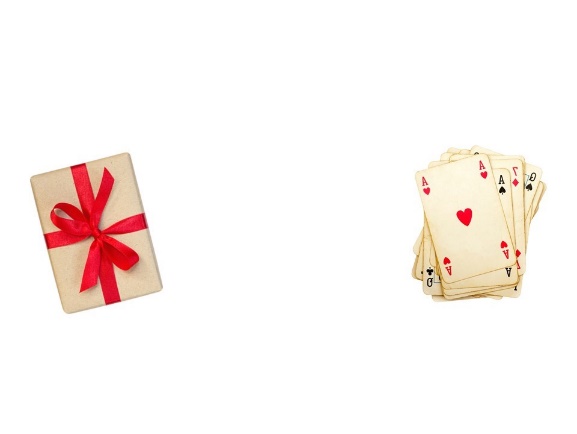 | |
| *English translation*  You are at a friend's birthday party. You congratulate her and give her your gift.   - Her sparkling eyes make you incredibly happy. (PI) - She is wearing a handful of paper streamers around her neck. (NI)   *German original*  Du bist auf der Geburtstagsfeier einer Freundin. Du gratulierst ihr und überreichst ihr dein Geschenk.   - Ihre strahlenden Augen machen dich unheimlich glücklich. (PI) - Sie trägt eine Handvoll Luftschlangen um den Hals herum. (NI) | *English translation*  You are with friends on a game night. It is your turn and you draw a new card*.*   - Your surprise victory brings you great joy. (PI) - The round is one of many in this game. (NI)   *German original*  Du bist bei Freunden auf einem Spieleabend. Du bist an der Reihe und ziehst eine neue Karte.   - Dein überraschender Sieg bereitet dir große Freude. (PI) - Die Runde ist eine von sehr vielen in diesem Spiel. (NI) |
| **Image Pair 11** | |
| 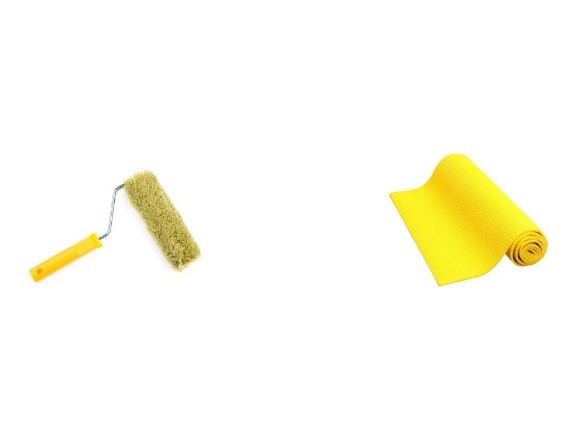 | |
| *English translation*  You are painting your home. You set down the paint roller and look at the wall.   - The refreshing color change makes you unspeakably happy. (PI) - The wall is wallpapered with ordinary woodchip wallpaper. (NI)   *German original*  Du bist zuhause am Streichen. Du setzt die Farbrolle ab und betrachtest die Wand.   - Der erfrischende Farbwechsel macht dich unsagbar glücklich. (PI) - Die Wand ist mit einer gewöhnlichen Raufasertapete tapeziert. (NI) | *English translation*  You are at home doing yoga. You sit down on the yoga mat and close your eyes.   - Your growing calm allows you to relax completely. (PI) - No other noises can be heard in the room. (NI)   *German original*  Du bist zuhause und machst Yoga. Du setzt dich auf die Yogamatte und schließt die Augen.   - Deine wachsende Ruhe lässt dich vollkommen entspannen. (PI) - In dem Raum sind keine anderen Geräusche zu hören. (NI) |
| **Image Pair 12** | |
| 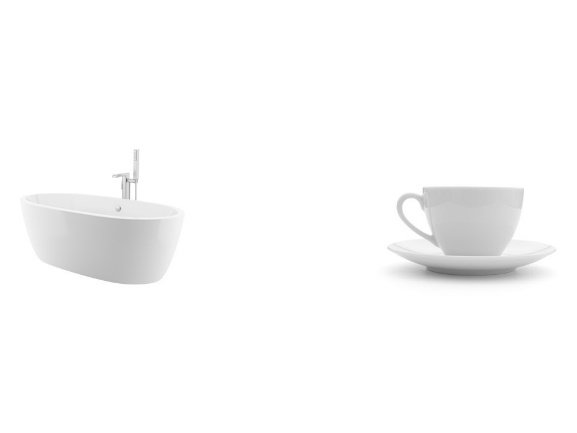 | |
| *English translation*  You are home running a bath. You climb into the bathtub and sit back.   - The warm water lets you relax completely. (PI) - The water surface is covered by a layer of foam. (NI)   *German original*  Du bist zuhause und lässt dir ein Bad ein. Du steigst in die Badewanne und lehnst dich zurück.   - Das warme Wasser lässt dich vollkommen entspannen. (PI) - Die Wasseroberfläche ist von einer Schaumschicht bedeckt. (NI) | *English translation*  You are home making coffee. You get a cup and pour it with coffee.   - The first sip is pure pleasure for you. (PI) - The coffee is a dark brown color. (NI)   *German original*  Du bist zuhause und machst Kaffee. Du holst dir eine Tasse und schenkst sie mit Kaffee ein.   - Der erste Schluck ist purer Genuss für dich. (PI) - Der Kaffee hat eine dunkel braune Farbe. (NI*)* |
| **Image Pair 13** | |
| 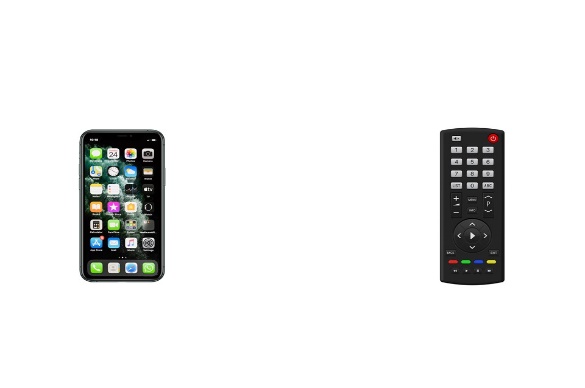 | |
| *English translation*  You are at home calling a friend. You pick up your cell phone and dial his number.   - You are so happy to hear his voice again. (PI) - The waiting signal sounds at regular intervals. (NI)   *German original*  Du bist zuhause und rufst einen Freund an. Du nimmst dein Handy in die Hand und wählst seine Nummer.   - Du freust dich riesig seine Stimme wiederzuhören. (PI) - Das Wartezeichen ertönt in regelmäßigen Abständen. (NI) | *English translation*  You are at home and watching a series. You grab the remote control and select the next episode.   - The theme tune gets you in sheer excitement. (PI) - A loading bar shows how far the episode has loaded. (NI)   *German original*  Du bist zuhause und schaust eine Serie. Du greifst nach der Fernbedienung und wählst die nächste Folge aus.   - Die Titelmelodie versetzt dich in schiere Begeisterung. (PI) - Ein Ladebalken zeigt an wie weit die Folge geladen ist. (NI) |
| **Image Pair 14** | |
| 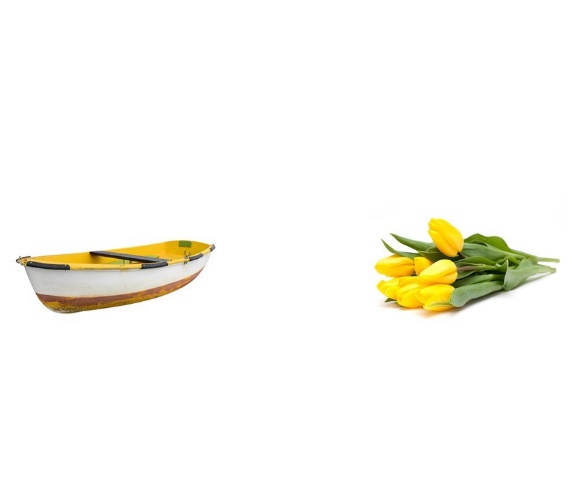 | |
| *English translation*  You are at the lake fishing. You let the rowboat drift and begin to wait.   - The tranquility of nature is a true pleasure for you. (PI) - Tall reeds grow along the edge of the lake. (NI)   *German original*  Du bist am See und angelst. Du lässt das Ruderboot treiben und fängst an zu warten.   - Die Ruhe der Natur ist ein einzig wahrer Genuss für dich. (PI) - Am Uferrand des Sees entlang wachsen hohe Schilfrohre. (NI) | *English translation*  You are in a field picking flowers. You take a ribbon out of your pocket and tie them into a bouquet.   - The bright colors make you incredibly happy. (PI) - The ends of the ribbon flutter slightly in the wind. (NI)   *German original*  Du bist auf einem Feld und pflückst Blumen. Du holst ein Bändchen aus der Tasche und bindest sie zu einem Blumenstrauß.   - Die bunten Farben machen dich unheimlich glücklich. (PI) - Die Enden des Bändchens flattern leicht im Wind. (NI) |
| **Image Pair 15** | |
| 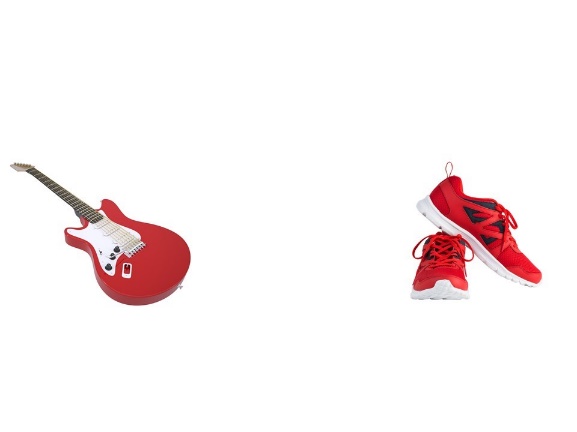 | |
| *English translation*  You are at home and you play the guitar. You put your fingers on the guitar strings and strum them.   - With every move you become more immersed in the music. (PI) - A metronome gives the right beat. (NI)   *German original*  Du bist zuhause und spielst Gitarre. Du legst die Finger auf die Gitarrensaiten und schlägst sie an.   - Mit jedem Griff gehst du mehr in der Musik auf. (PI) - Ein Taktmesser gibt dabei den richtigen Takt vor. (NI) | *English translation*  You are going for a jog outside. You want to test your running shoes and are running through a forest.   - You are very euphoric about the ease of your movements. (PI) - There are many large deciduous trees along the route. (NI)   *German original*  Du bist draußen eine Runde joggen. Du möchtest deine Laufschuhe testen und läufst dafür durch einen Wald.   - Du bist ganz euphorisch über die Leichtigkeit deiner Bewegungen. (PI) - An der Strecke entlang befinden sich viele große Laubbäume. (NI) |

COPYRIGHT STATEMENT

All images and imagery scripts are subject to copyright and not for use by other parties. All images in this study were used under license from Shutterstock.com.

**B: Results**

**Table B1.** Questionnaire descriptives (*N* = 54)

| **Questionnaire** | ***M* (*SD*)** |
| --- | --- |
| DASS-21 |  |
| Total score | 31.33 (8.15) |
| Depression subscale | 10.19 (2.98) |
| Anxiety subscale | 9.19 (2.73) |
| Stress subscale | 11.96 (3.83) |
| PANAS |  |
| Positive Affect subscale | 3.34 (.61) |
| Negative Affect subscale | 1.25 (.24) |
| PSI-Q | 8.73 (1.32) |
| SUIS | 3.41 (.70) |
